# In This Issue

**Published:** 2000

## Introduction

The National Institute on Alcohol Abuse and Alcoholism (NIAAA) is committed to making research findings accessible to a broad audience. We do this in many ways, including general and specialized brochures, pamphlets, manuals, clinical bulletins, and through our Web site. Because we believe strongly that creditable, science-based information is our best defense against the ages-old stigma associated with alcoholism and other alcohol-related problems, we are always looking for new opportunities and better ways to share science with our many audiences. The recent release of the *Tenth Special Report to the United States Congress on Alcohol and Health* has presented such an opportunity.

The triennial *Reports to the United States Congress on Alcohol and Health* chart the progress made by alcohol research toward understanding, preventing, and treating alcohol abuse and alcoholism. They cover a full range of topics in alcohol biological and behavioral research. As such, the Special Reports serve as primary sources of information on alcohol-related problems for a variety of audiences, including researchers, public policy makers, clinicians, educators, and the general public. The full *Tenth Special Report* is available on NIAAA’s Web site at http://www.niaaa.nih.gov. We are delighted to also be able to share the many exciting new findings from this report through this *Alcohol Research & Health* in a summary format that will be useful for many different audiences in many different ways.

As we proceed through this new era, I believe that we will see much progress toward developing highly effective ways to prevent and treat alcohol-related problems. We will continue to look for venues to share the excitement of this progress and its hope to the individuals, families, and society who must bear the burden of alcohol abuse and alcoholism.

Enoch Gordis, M.D., Director, National Institute on Alcohol Abuse and Alcoholism, National Institutes of Health

## ALCOHOL RISKS AND BENEFITS

Forty-four percent of the adult U.S. population (aged 18 and over) are current drinkers who have consumed at least 12 drinks in the preceding ye a r. Most people who drink do so safely. A small percentage of people drink heavily, however, a practice that often has serious repercussions for the drinker, and his or her family, friends, coworkers, and the community. The research reviewed in this article represents a wide spectrum of approaches to understanding the causes and consequences of alcohol use and abuse over the course of the lifespan. These research findings can help shape the efforts of communities to reduce the negative consequences of alcohol consumption, assist health practitioners in advising alcohol consumers, and help people make informed decisions about the positive and negative aspects of drinking. (pp. 5–11)

## ALCOHOL, THE BRAIN, AND BEHAVIOR: MECHANISMS OF ADDICTION

The mechanisms that promote the development of alcoholism occur primarily in the brain. This article reviews recent studies that shed light on the motivation to seek and consume alcohol despite attempts to achieve abstinence. For example, the brain of a heavy drinker undergoes changes (i.e., adaptations) in order to continue functioning in the presence of alcohol. Some of these changes may become permanent, leading to persistent discomfort that may lead to relapse to drinking long after a person has stopped drinking. A better understanding of the complex, multilevel interactions among the brain chemicals that underlie these changes will assist alcoholism prevention and treatment efforts. (pp. 12–15)

## WHY DO SOME PEOPLE DRINK TOO MUCH?

A person’s risk for developing alcohol-related problems and alcoholism is influenced by both genetic and environmental (i.e., psychosocial) factors. This article explores methods being used to identify the genes that convey a risk for alcoholism. For example, quantitative trait loci (QTL) mapping in rats has helped identify several genes that may influence the animal’s preference for alcohol. The article also describes the plethora of psychosocial factors that contribute to the risk for alcoholism, including a family history of alcoholism, parental psychopathology, family environment, developmental processes, and motivations for alcohol consumption (e.g., drinking to reduce stress). (pp. 17–26)

## MEDICAL CONSEQUENCES OF ALCOHOL CONSUMPTION

As reviewed in this article, there is hardly a system of the body that is not affected by alcohol. Alcoholic liver disease is the second leading reason for liver transplantation. Alcohol-induced brain disorders such as Korsakoff’s psychosis can produce devastating amnesia or dementia. Impairments of the immune system lead to recurrent infections among alcoholics. Alcohol also has been associated with bone disorders and potentially implicated in the development of breast cancer. Moderate drinking (two drinks per day for men and one drink for women) may have some protective effect against coronary artery disease. However, alcohol also may have serious harmful effects on the heart and other organ systems. This article reviews both the harmful and benefical effects of alcohol on the body. (pp. 27–31)

## PRENATAL EXPOSURE TO ALCOHOL

Maternal alcohol consumption during pregnancy can lead to serious birth defects. The most severe of these conditions is fetal alcohol syndrome (FAS), which is characterized by growth deficiency; certain facial features; and deficits in various areas of intellectual functioning. Less extensive, but equally serious consequences of prenatal alcohol exposure include alcohol-related neurodevelopmental disorder (ARND) and physical abnormalities termed alcohol-related birth defects (ARBD). As reported in this article, children with FAS show widespread and severe brain damage, particularly in the cerebellum and the basal ganglia, which impairs brain function and contributes to intellectual deficits. The timing and pattern (e.g., binge drinking) of maternal drinking during pregnancy influences the type and severity of fetal damage. A number of mechanisms have been implicated in alcohol’s detrimental effect on the fetus. Those mechanisms and a number of approaches to prevent FAS are presented. (pp. 32–41)

## LATEST APPROACHES TO PREVENTING ALCOHOL ABUSE AND ALCOHOLISM

Scientists and policymakers have explored numerous strategies to prevent alcohol abuse and dependence as well as the adverse social, legal, and medical consequences of alcohol use. Many of these efforts have focused on reducing alcohol-impaired driving and the associated injuries and fatalities. As reported in this article, such efforts have included general deterrence laws (e.g., reduced minimum legal drinking age, administrative license revocation, and lower legal limits for blood alcohol concentrations), measures targeted at repeat offenders, and measures to control alcohol availability (e.g., increased taxes and decreased numbers of establishments selling alcohol). (pp. 42–51)

## RESEARCH REFINES ALCOHOLISM TREATMENT OPTIONS

Every day, more than 700,000 people in the United States receive alcoholism treatment. This article describes the range of treatment options available. For example, patients who are at risk for or who are experiencing alcohol problems can benefit from brief interventions delivered by their primary care physicians. Alcohol-dependent patients require more extensive treatment with either psychological or pharmacological approaches. Findings from studies assessing the effects of matching patients to specific treatments based on patient characteristics also are presented. Professional treatment combined with a 12-step approach (e.g., Alcoholics Anonymous) appears to be the most effective approach for many patients. Pharmacological treatment with opiate antagonists (e.g., naltrexone) and other agents can substantially improve abstinence after therapy, even in patients with co-occurring psychiatric disorders, such as depression. (pp. 53–61)

## ECONOMIC ANALYSIS AIDS ALCOHOL RESEARCH

Economic research contributes to our understanding of alcohol use and the prevention and treatment of alcohol-related problems in several ways. This article reviews three areas in which the tools of economic analysis have produced significant insights in recent years. First, economic researchers have analyzed the effects of beverage prices and taxation on alcohol consumption and on adverse consequences associated with alcohol use. Second, analyses of the costs and cost-effectiveness of treatment for alcohol use disorders have provided insight into the long-term costs and benefits of alternative approaches to alcoholism treatment. Finally, studies have incorporated economic techniques in estimating the overall magnitude of the burden placed on society by the misuse of alcoholic beverages. (pp. 62–71)

